# Inter‐annual variation in amphibian larval interspecies interactions

**DOI:** 10.1002/ece3.10221

**Published:** 2023-07-04

**Authors:** Jessica Ford, David M. Green

**Affiliations:** ^1^ Redpath Museum McGill University Montreal Quebec Canada

**Keywords:** competition, inter‐annual variation, tadpoles, toads

## Abstract

The outcomes of species interactions can vary by life stage, year, and surrounding environmental conditions. Amphibian species are expected to compete most strongly during their tadpole stage when they exist in the highest densities. Changes in arrival timing, surrounding aquatic communities, and yearly conditions could all affect the outcome of larval competition. In Long Point, Ontario, the Fowler's toad (*Anaxyrus fowleri*) is at the northern edge of its range and overlaps with the more common American toad (*Anaxyrus americanus*). Both species breed in ponds that encounter high inter‐annual variation. To determine whether these species compete strongly, and if this effect was replicated across multiple years, we raised both species as tadpoles together and, apart, in mesocosms in 2018 and 2021. We measured survivorship to, weight at, and time to metamorphosis for both species in both years. We determined that the presence of American toad tadpoles consistently had a detrimental effect on Fowler's toad tadpoles, even though this effect presented itself differently across years. Our study suggests that competitive exclusion by American toads could be occurring at the edge of the Fowler's toad's range. This study further demonstrates the importance of studying communities across multiple years to understand the full scope of species interactions.

## Graphical abstract

The outcome of interspecific competition between two species of toad tadpoles changed between years. In 2018, emerging Fowler's toadlets had poor survivorship but a greater weight when raised with American toad tadpoles, while in 2021 Fowler's toadlets had good survivorship but a lower weight when raised with American toad tadpoles. Inter‐annual variation should be taken into account in order to capture the full scope of species interactions.

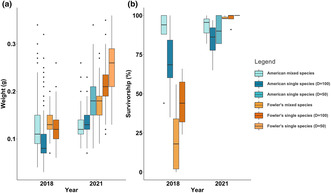

## 1 INTRODUCTION

According to the competitive exclusion principle, two species with identical niches cannot coexist, as one species will eventually drive the other to extinction (Hardin, 1960; Hening & Nguyen, 2020; Levin, 1970). Species are more likely to compete strongly if their niche overlap is large, or if they are competing for the same food resource (Hardin, 1960; Hening & Nguyen, 2020; Levin, 1970). Ecologically similar species will thus compete strongly when habitat or food availability is reduced, or where the edges of their ranges overlap, as has been shown in sea birds in marine environments (Bonnet‐Lebrun et al., 2021) and foxes in terrestrial environments (Elmhagen et al., 2017; Gosselink et al., 2003).

However, which species dominates may not be predictable, as many factors can alter the outcome of interspecies interactions, including temporal variation (Hutchinson, 1957; Rudolf, 2019). Non‐equilibrium conditions caused by temporal variation can allow ecologically similar species to have competitive advantages at different times, enabling coexistence where it would not occur otherwise (Grainger et al., 2019; Hening & Nguyen, 2020). Accounting for temporal changes in species interactions over time is thus essential for understanding species coexistence and community structure (Angert et al., 2009; Chesson, 2000; Rudolf, 2019). This temporal variation, or inter‐annual variation, is often caused by year effects, such as changes in weather and environmental conditions (Dakos et al., 2009; Werner et al., 2020). Year effects can exert profound impacts on community assembly, community composition, and ecological dynamics (Rudolf, 2018; Werner et al., 2020). Despite the importance of inter‐annual variation, changes in the outcome of species interactions between years are rarely studied or considered (Rudolf, 2018, 2019; Werner et al., 2020).

Small aquatic ecosystems, such as ponds, can be heavily affected by inter‐annual variation in precipitation, as this can change hydroperiod and pond size, with ensuing effects on the interactions between inhabitants (Reinhardt et al., 2015). Ponds are dynamic and often ephemeral habitats that support high biodiversity and serve as refuge sites for many species (Hill et al., 2021; Reinhardt et al., 2015). Inter‐annual variation in species composition has been observed in ponds even in successive years, affecting the phenology of phytoplankton community blooms and their interactions with keystone herbivores in the zooplankton community (Winder & Schindler, 2004). Temporal variation in climatic conditions can also change the establishment times of invertebrate communities in ponds and alter their interactions with larval amphibian communities, as well as how the larval amphibians interact with one another (Reinhardt et al., 2015). This change in larval amphibian communities can, in turn, have profound feedback effects on the surrounding algal and zooplankton communities (Arribas et al., 2014; Buck et al., 2012; Hamilton et al., 2012). Understanding the impacts of inter‐annual variation in relation to larval amphibian interactions, such as competition, is thus essential to comprehending the ecology of small aquatic ecosystems.

The tadpoles of pond‐breeding anurans are important components of many small aquatic ecosystems (Arribas et al., 2014; Buck et al., 2012; Hamilton et al., 2012). Competition between closely related tadpole species can influence breeding site selection (Buxton & Sperry, 2017), the structure of tadpole communities (Faragher & Jaeger, 1998; Stein et al., 2017), and the success of larval development (Wilbur, 1987). Tadpoles within ponds may be at very high densities, and often share the same primary food source of periphyton (Connelly et al., 2008; Hamilton et al., 2012; Wood & Richardson, 2010), facilitating strong competition between species (Altwegg, 2003; Gazzola & Buskirk, 2015; Pechmann, 1995; Wilbur, 1980). Tadpoles are ephemeral, and the timing of adult breeding in ponds can change each year, altering competition dynamics (Alford & Wilbur, 1985; Lawler & Morin, 1993; Rudolf, 2018). Furthermore, the ponds in which tadpoles live will change in response to weather conditions (Florencio, Burraco, et al., 2020; Florencio et al., 2009; Reinhardt et al., 2015; Rudolf, 2018), which can affect aquatic community structure and impact the outcomes of interspecies competition. Tadpoles, being the larval form of amphibians, do not have other forms of competition such as sexual competition for mates, which may complicate studies on resource competition. The effects of larval competition, and the resulting fitness of metamorphs, can be determined by time to metamorphosis, survivorship through metamorphosis, and the weight of metamorphs as they emerge (Bardsley & Beebee, 2001; Dash & Hota, 1980; Stein et al., 2017). These metrics can be measured in a relatively straightforward manner, making the model system of tadpole communities in ponds valuable for studying competition and inter‐annual variation (Bardsley & Beebee, 2001).

Throughout much of eastern North America, Fowler's Toads, *Anaxyrus fowleri*, overlap in range with American Toads, *Anaxyrus americanus*, including in our study site of Long Point, Ontario. As adults, Fowler's toads tend to be associated with sand dune habitats whereas American toads are a generalist species (Petranka et al., 1994). However, both species will breed in shallow, sandy, nutrient‐poor ponds and have been known to breed in the same ponds (Green, 1982) at locations where their ranges overlap, and even hybridize (Green, 1982; Green & Parent, 2003; Zweifel, 1968). Under these circumstances, competition between tadpoles of these two species is highly probable.

American toad tadpoles may have an advantage over Fowler's toad tadpoles when they co‐occur. As American toads breed roughly 2 weeks earlier in the spring than Fowler's toads (Volpe, 1955), their tadpoles are likely to be larger and at a later developmental stage than any Fowler's toad tadpoles present in the same pond. Because of this priority effect, we expected that American toad tadpoles would outcompete Fowler's toad tadpoles for food resources, consisting mainly of periphyton (Connelly et al., 2008; Hamilton et al., 2012; Wood & Richardson, 2010), possibly causing American toad tadpoles to metamorphose more quickly, with higher survivorship (Alford & Wilbur, 1985; Woodward, 1987).

Under stable environmental conditions, niche partitioning due to the habitat preferences of breeding adult toads may occur to reduce competition among the different species of tadpoles. However, these toads do not live in stable conditions. The landscape of Long Point is a highly dynamic sand dune and marshland environment that is heavily influenced by fluctuating water levels and storm‐driven waves from Lake Erie (Hebb, 2003). Thus, the two species may instead co‐exist in an unstable environment subject to year effects. To test between these alternative scenarios, it is first necessary to establish whether the American toad tadpoles have a detrimental effect on Fowler's toad tadpoles, consistent with competitive exclusion. If competitive exclusion is likely to occur with these toad species, then the tadpoles of one species should have a detrimental effect on the tadpoles of the other species. The disadvantaged species, which we expect to be the Fowler's toad tadpoles, should exhibit lower survivorship, reduced size at metamorphosis, and prolonged time to metamorphosis when raised in mesocosms with American toad tadpoles compared with when raised alone. However, if inter‐annual variation alters the outcome of such competitive interactions, then repetition of the experiment in different years could generate significantly different results.

## 2 METHODS

To assess the result of competitive interactions and year effects, we raised American Toad and Fowler's Toad tadpoles in Rubbermaid© structural foam cattle watering tanks, which we used as outdoor mesocosms (Ford & Green, 2021), during 2018 and 2021 at Long Point Provincial Park, Ontario. The mesocosms measured 63.50 cm L × 78.74 cm W × 134.6 cm H and were covered with a shade cloth to prevent insects and other animals from entering. The experiment was initially conducted in 2018 but not repeated until 2021 due the late emergence of Fowler's Toads at the site in 2019 and closure of access to the site in 2020 due to the COVID‐19 pandemic. The tadpoles we used were hatched from toad eggs collected from clutches laid in nearby local ponds or from egg clutches laid by amplectic pairs that we placed in a specialized breeding tank for 24 h. In 2018, we collected three clutches of American toad eggs from a natural pond on May 3rd, and one clutch of Fowler's toad eggs was collected from an amplectic pair on May 10th. In 2021, two clutches of American toad eggs were collected from natural ponds on May 4th and May 16th and one clutch of Fowler's toad eggs was collected from an amplectic pair on May 19th. One clutch of Fowler's toad eggs were located each year as Fowler's toads are endangered in Canada and did not have any large breeding choruses in either 2018 or 2021.

We obtained data on air temperature (maximum, minimum, and mean daily temperatures, in °C) from the Government of Canada (https://climate.weather.gc.ca) for the Long Point Weather Station (Latitude 42.53°N|Longitude 80.05°W, approximately 28 km from our study site). Rainfall (total monthly precipitation, in mm, and percent average precipitation) data was sourced from Agricorp for the hamlet of Charlotteville, Ontario (now known as Walsh, Ontario) approximately 20 km from our study site. We measured water temperature (°C) between noon and 2:00 p.m. each day using an EcoSense oxygen probe (YSI, DO200) in 2018 and a HANNA multiparameter probe (HANNA Instruments, HI98194) in 2021. We assessed differences in air and water temperature across years using unpaired *t*‐tests.

Each year, we established mesocosms in which to raise tadpoles according to a standardized protocol (Ford & Green, 2021). We monitored the mesocosms continuously for changes in ammonia, nitrate, nitrite, oxygen, and signs of mortality (Ford & Green, 2021). To ensure uniformity and prevent a spike in ammonia or nitrate from affecting tadpole success, we conducted partial, 20% water changes when ammonia or nitrate were above 0 ppm. Only two such water changes were needed in 2018, and none were required in 2021. We also set up additional mesocosms to house eggs and hatchling tadpoles, or for use during water changes. Any tadpoles remaining in additional mesocosms after the addition of tadpoles to experimental mesocosms were released at the point of origin (approximately 2.15 km from the mesocosm).

In 2018, we used 17 mesocosms to house tadpoles: six with 100 American toad tadpoles, six with 100 Fowler's toad tadpoles, and five with a mixture of 50 tadpoles of each species. In 2021, we used 20 mesocosms to house tadpoles: four with 50 Fowler's toad tadpoles, four with 100 Fowler's toad tadpoles, five with 50 American toad tadpoles, four with 100 American toad tadpoles, and three with 50 tadpoles of each species. The number of replicates varied between years as we had different amounts of experimental groups within the 30 mesocosms worked within each year.

When toadlets reached developmental stage 42 (Gosner, 1960) we removed them from the mesocosms, weighed them individually, recorded the presence or absence of a tail, and released them at point of origin. We determined time to metamorphosis by recording the first day a toadlet was found in each mesocosm. We tested for significant differences in weight at metamorphosis and time to metamorphosis between single and mixed‐species mesocosms using unpaired *t‐*tests.

We calculated survivorship as the number of metamorphic toadlets of a species ultimately collected from a mesocosm compared with the number of tadpoles of that species originally placed in that mesocosm. To test whether tadpoles of one species had a detrimental effect on tadpoles of the other species, we used Generalized Linear Mixed Models (GLMMs). Our GLMMs used the response variable *tadpole survivorship*, explanatory variable *tank kind* (categorical, *n* = 2 in 2018 and *n* = 3 in 2021; levels in 2018: single‐species, mixed‐species; levels in 2021: single‐species, single‐species half density, mixed‐species) and random effect variables *tank number* (*n* = 17 in 2018 and *n* = 20 in 2021). We ran separate models for the Fowler's toad dataset and American toad dataset in both years. We tested the data for overdispersion and found Poisson distribution to be optimal (R package: lme4). If the Poisson distribution was overdispersed (indicated by the quotient of residual deviance by degrees of freedom being much greater than one), we tested again using quassipoisson and negative binomial distribution models. Akaike's information criterion was used to determine the model that best fit the data (R package: DHARMa).

To determine whether the year of the study, as well as the composition of mesocosms, had a significant effect on tadpole survivorship, we ran another GLMM. This GLMM had the response variable *tadpoles survivorship*, two explanatory variables, *tank kind* (this time combining 2018 and 2021, categorical, *n* = 3, single‐species, single‐species half density, mixed‐species) and *year* (categorical, *n* = 2, levels: 2018 and 2021), as well as the random effect *tank number* (*n* = 37).

All statistics were performed in R v. 4.1.1 (R Core Team, 2021).

## 3 RESULTS

Yearly conditions varied between 2018 and 2021; 2018 was comparatively warm and dry, whereas 2021 was noticeably cooler and wetter (Table 1). The average daily maximum water temperature in the mesocosms was generally higher in 2018 than in 2021. In 2018, May, July, and August were 6.8, 4.6, and 4.4°C warmer, respectively, than in 2021. In 2021, June was marginally warmer than 2018, but by only 0.8°C. The average daily minimum air temperature was also slightly warmer in 2018 than in 2021. A *t*‐test revealed that mean air temperature was significantly warmer in 2018 than in 2021 (*t*‐test: *t* = 5.1194, df = 57 *p* < .0001). Mesocosm water temperatures were significantly warmer in 2018 in May (*t*‐test: *t* = 6.3133, df = 298, *p* < .0001), July (*t*‐test: *t* = 7.0836, df = 988, *p* < .0001), and August, (*t*‐test: *t* = 24.0248, df = 67, *p* < .0001), and significantly warmer in 2021 in June (*t*‐test: *t* = 4.41477, df = 794, *p* < .0001). However, it should be noted that while these differences were significant, they represent a difference of less than 4°C, and the large sample sizes may have resulted in an overpowered *t*‐test.

**TABLE 1:** Average air temperature, mesocosm water temperature, and rainfall data for Long Point, Ontario, in 2018 and 2021.

Year	Month	Average daily max air temp (°C) ± SD	Average daily mean air temp (°C) ± SD	Average daily min air temp (°C) ± SD	Average daily mean water temp (°C) ± SD	Total rainfall (mm)	Percent of normal rainfall (%)
2018	May	19.4 ± 4.7	14.7 ± 4.0	9.8 ± 4.0	23.9 ± 0.5	69.8	80
	June	21.5 ± 3.1	18.9 ± 2.6	16.4 ± 2.5	21.6 ± 2.9	93.4	120
	July	25.6 ± 2.2	23.2 ± 1.9	20.8 ± 2.0	27.9 ± 11.5	95.4	60
	August	24.8 ± 1.8	23.0 ± 1.5	21.2 ± 1.6	28.8 ± 0.7	83.0	120
2021	May	18.3 ± 5.6	12.5 ± 5.1	6.6 ± 5.0	17.1 ± 4.3	40.2	60
	June	25.1 ± 3.3	20.0 ± 3.2	15.0 ± 3.7	22.4 ± 2.5	158.4	120
	July	24.8 ± 2.2	20.6 ± 2.0	16.4 ± 2.1	23.3 ± 1.7	156.4	160
	August	28.3 ± 2.7	24.1 ± 2.7	19.9 ± 3.5	24.4 ± 0.7	74	–

*Note*: Standard deviation is not shown for rainfall data as only total rainfall values were collected by Agricorp.

The year 2021 had notably more rainfall than 2018, especially in July, which accumulated 160% of the average rainfall in 2021 but only 60% of the average rainfall in 2018, an approximately 2.7 times increase. This was accompanied by many intense rainstorms, sometimes lasting for days at a time. In 2021, June and July had 65.0 and 61.0 mm more rainfall, respectively, than in 2018. In May and August, however, there was 29.6 and 9.0 mm, respectively, more rain in 2018 than in 2021.

**FIGURE 1:**
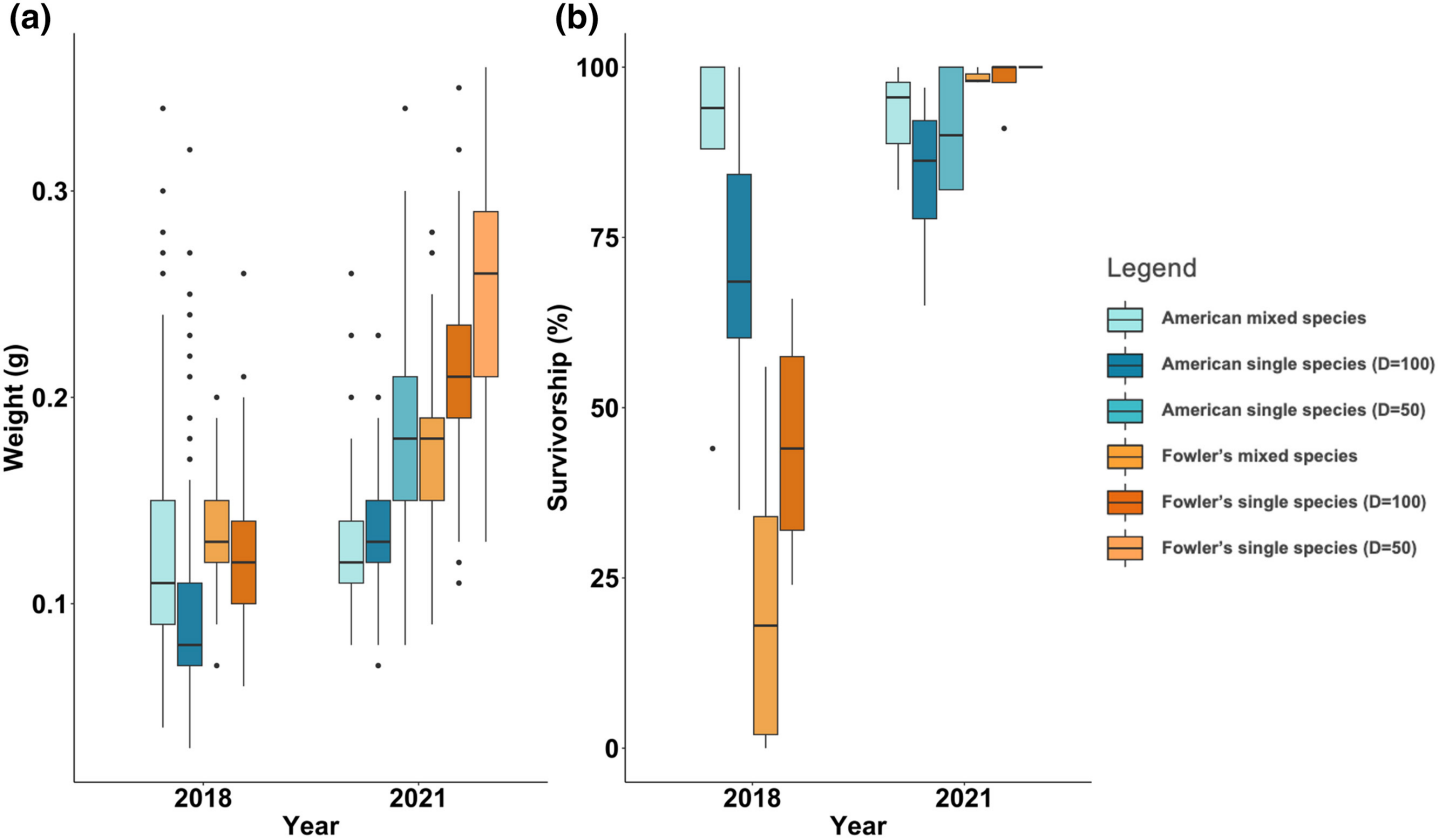
(a) Average survivorship at metamorphosis of American toad tadpoles and Fowler's toad tadpoles in single‐species and mixed‐species mesocosms in 2018 and 2021. (b) Average weight to metamorphosis of American toad tadpoles and Fowler's toad tadpoles in single‐species and mixed‐species mesocosms in 2018 and 2021.

**FIGURE 2:**
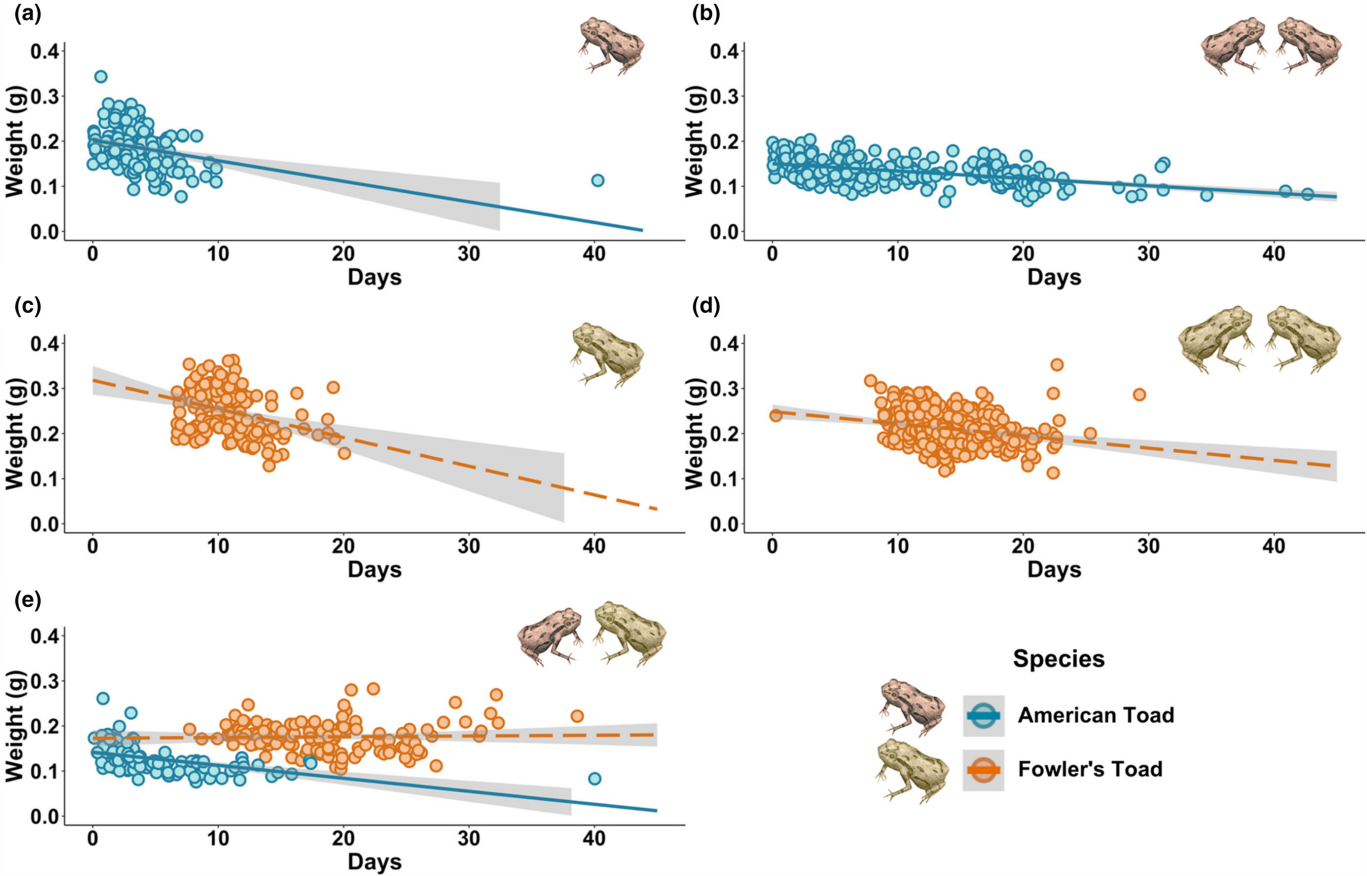
Weight of toadlets emerging from each experimental group of mesocosms in 2021, starting from the first day of toadlet emergence (June 16th). Each point represents an individual toadlet. (a) Single‐species American toad tadpole mesocosms with a density of 50 tadpoles. (b) Single‐species American toad tadpole mesocosms with a density of 100 tadpoles. (c) Single‐species Fowler's toad tadpole mesocosms with a density of 50 tadpoles. (d) Single‐species Fowler's toad tadpole mesocosms with a density of 100 tadpoles. (e) Mixed‐species American toad and Fowler's toad tadpole mesocosms with a density of 100 tadpoles (50 of each species). *Illustrations by Jessica Ford*.

Fowler's toad tadpoles were negatively affected by the presence of American toad tadpoles in both 2018 and 2021, with the fitness parameters of time to, weight at, and survivorship to metamorphosis differing in the presence of competitors, but not in the same ways each year (Table 2; Figure 1). In 2018, while there was no significant difference between time to metamorphosis between American toadlets in single‐species or mixed‐species mesocosms, there was a significant difference between the time to metamorphosis of Fowler's toadlets in single‐species and mixed‐species mesocosms (Table 2). Fowler's toadlets took significantly longer to metamorphose in mixed‐species mesocosms than in single‐species mesocosms (*t*‐test: *t* = 3.934, df = 9, *p* = .0034). In 2021, although not significant, Fowler's toadlets from mixed‐species mesocosms still emerged later than in single‐species mesocosms with an initial density of either 50 or 100 tadpoles, with an average of 42.23 (±4.90, *n* = 3) days compared with 36.3 (±0.60, *n* = 3) and 38.0 (±0.80, *n* = 4) days, respectively (Table 2). As these data were analyzed using an unpaired *t*‐test, the high variance in time to metamorphosis of Fowler's toadlets in mixed‐species mesocosms may account for the lack of a significant difference despite the trend. Additionally, Fowler's toadlets emerging from mixed‐species mesocosms in 2021 only began metamorphosis after most of the American toadlets had left the mesocosms and did not decrease in weight at metamorphosis over time, which contrasts with all other mesocosms, where toadlet weight at metamorphosis, regardless of species, decreased over time (Figure 2). Similar data for 2018 is unavailable as too few Fowler's toad tadpoles survived to metamorphosis in the mixed‐species mesocosms to observe any trends. American toadlets in mixed‐species mesocosms and single‐species mesocosms showed no significant difference in time to metamorphosis in either 2018 or 2021.

**TABLE 2:** Average survivorship of American toad tadpoles and Fowler's toad tadpoles to metamorphosis in single‐species and mixed‐species mesocosms in 2018 and 2021.

Year	Species present	No. of mesocosms	Tadpoles per mesocosm	% Survivorship per mesocosm ± SD	Toadlet weight		Time to metamorphosis	
					Mean (g) ± SD	*n* (individuals)	Mean (days) ± SD	*n* (mesocosms)
2018	American toad	6	100	70.0 ± 23.20	0.09 ± 0.06	413	47.1 ± 2.93	6
	Fowler's toad	6	100	45.0 ± 17.09	0.12 ± 0.03	232	40.3 ± 2.50	6
	Mixed species: American toad	5	50	85.0 ± 11.78	0.12 ± 0.05	213	46.4 ± ± 2.07	5
	Mixed species: Fowler's toad	5	50	22.0 ± 11.72	0.14 ± 0.03	55	48.3 ± 4.19	5
2021	American toad	5	50	90.8 ± 9.01	0.18 ± 0.03	227	32.0 ± 0.00	5
	American toad	4	100	83.5 ± 13.77	0.14 ± 0.01	334	32.3 ± 0.50	4
	Fowler's toad	3	50	100 ± 0.00	0.25 ± 0.05	150	36.3 ± 0.60	3
	Fowler's toad	4	100	95.5 ± 9.00	0.21 ± 0.02	382	38.0 ± 0.80	4
	Mixed species: American toad	3	50	92.6 ± 9.45	0.13 ± 0.01	139	32.0 ± 0.00	3
	Mixed species: Fowler's toad	3	50	98.0 ± 2.00	0.17 ± 0.02	147	42.23 ± 4.90	3

The weight of emerging American and Fowler's toadlets also varied between treatment and year (Table 2). In 2018, Fowler's toadlets weighed significantly more in mixed‐species mesocosms compared with single‐species mesocosms (*t*‐test: *t* = 3.996, df = 56.818, *p* < .001). Similarly, American toadlets emerging from mixed‐species mesocosms weighed significantly more than their single‐species counterparts in 2018 (*t*‐test: *t* = −7.835, df = 331.52, *p* < .001). In 2021, however, Fowler's toadlets emerging from mixed‐species mesocosms weighed significantly less than Fowler's toadlets from single‐species mesocosms (Table 2) with an initial density of 50 tadpoles (*t*‐test: *t* = 15.804, df = 282.99, *p* < .0001) and 100 tadpoles (*t*‐test: *t* = 10.622, df = 250.05, *p* < .0001). In addition, in 2021 American toadlets emerging from mixed‐species mesocosms also weighed significantly less than American toadlets emerging from single‐species mesocosms with an initial density of 50 tadpoles (*t*‐test: *t* = 14.716, df = 350.1, *p* < .0001) and American toadlets from mesocosms with an initial density of 100 tadpoles (*t*‐test: *t* = 2.177, df = 224.35, *p* = .0305).

Additionally, tadpole survivorship varied depending upon treatment and year (Table 2). In 2018, the average survivorship of Fowler's toad tadpoles to metamorphosis was significantly reduced in the presence of American toad tadpoles compared with mesocosms that contained only Fowler's toads (GLMM, Poisson distribution: *z* = 2.084, *p* = .0372). In 2021, however, survivorship of Fowler's toad tadpoles was not affected by the presence of American toad tadpoles, as all mesocosms produced high toadlet survivorship with values ranging from 95%–100%. There was also no difference in Fowler's toad survivorship between mixed‐species mesocosms and single‐species mesocosms at either a density of 100 tadpoles per mesocosm (GLMM, Poisson distribution: *z* = 0.122, *p* = .903) or a density of 50 tadpoles per mesocosm (GLMM, Poisson distribution: *z* = −0.087, *p* = .930) in 2021. In this model, American toad tadpole survivorship did not significantly differ between mixed‐species and single‐species mesocosms either in 2018 (GLMM, negative binomial distribution: *z* = 1.488, *p* = .147) or in 2021 at densities of 100 tadpoles per mesocosm GLMM, negative binomial distribution: (GLMM, negative binomial distribution: *z* = −0.943, *p* = .345) or 50 tadpoles per mesocosm (GLMM, negative binomial distribution: *z* value = −0.133, *p* = .894). All single‐species Fowler's toad and single‐species American toad mesocosms produced higher survivorship rates in 2021 than in 2018.

When running an additional GLMM involving the year of study, year also had a significant effect on the survivorship of Fowler's tadpoles (GLMM, quassipoisson equivalent distribution, *z* = 5.643, *p* < .001) but not on the survivorship of American toad tadpoles (GLMM, Poisson distribution, *z* = 1.600, *p* = .109). However, in the GLMM involving both tank kind and year, American toad tadpole survivorship was significantly higher in mixed‐species mesocosms than in single‐species mesocosms (GLMM, Poisson distribution, *z* = −2.121, *p* = .034).

## 4 DISCUSSION

While some species may exhibit competitive advantages over another, these roles are not always consistent (Rudolf, 2019). The outcome of species competition can be altered by phenological shifts and yearly conditions (Reinhardt et al., 2015; Rudolf, 2019). Our results indicate that year effects can create significant changes in the outcomes of species interactions. Specifically, Fowler's toadlet time to, weight at, and survivorship to metamorphosis when in the presence of American toadlets can vary significantly from 1 year to another (Table 3). In terms of competitive exclusion, Fowler's toadlets reared in the presence of American toad tadpoles demonstrated either lower relative fitness with decreased survivorship or lower weight at metamorphosis. Thus, it may be beneficial for Fowler's toads to avoid breeding in ponds with American toads. However, while American toad tadpoles always appear to have a detrimental impact on co‐occurring Fowler's toad tadpoles in experimental conditions, the appearance of this effect can be notably different between years.

**TABLE 3:** Summary of yearly condition trends and competition outcome for Fowler's toad tadpoles and American toad tadpoles in 2018 and 2021.

	2018	2021
Environmental variables		
Overall	Warmer and dryer	Cooler and wetter
Precipitation during May	Low	Low
Precipitation during June	High	High
Precipitation during July	Low	High
Fowler's toadlet fitness metrics compared to single‐species mesocosms		
Time to metamorphosis	Longer	Longer
Weight at metamorphosis	Higher	Lower
Survivorship	Lower	Same
American toadlet fitness metrics compared to single‐species mesocosms		
Time to metamorphosis	Same	Same
Weight at metamorphosis	Higher	Lower
Survivorship	Higher	Higher

The mechanism of the American toad tadpole's competitive advantage over Fowler's toad tadpoles is unclear. There are many ways tadpoles can interact with competitors, ranging from increased toxin production (Bókony et al., 2018) to trophic plasticity (Altig et al., 2007; Arribas et al., 2015; Caut et al., 2013) and predation or cannibalism (Polis et al., 1989). It is possible that, in 2018, American toad tadpoles scavenged on Fowler's toad tadpole remains or predated upon them directly, as Fowler's toads had very low survivorship in the mixed‐species mesocosms, but no deceased tadpoles were ever found in the mesocosms despite daily checks. Notably, this phenomenon only occurred in the mixed‐species mesocosms and was not density‐dependent or a crowding effect, as mixed‐species and single‐species mesocosms had the same density of tadpoles.

While many species of tadpoles, including Bufonid tadpoles, are often considered to be herbivorous, it has been suggested that many species may consume animal matter such as zooplankton (Hamilton et al., 2012; Khan, 2014) and have trophic plasticity (Altig et al., 2007; Arribas et al., 2015; Caut et al., 2013). In 2018, American toad metamorphs were larger from mixed‐species mesocosms than single‐species mesocosms, possibly from predating or scavenging the remains of Fowler's toad tadpoles. It has previously been noted that American toad tadpoles that scavenge on the remains of deceased conspecifics tend to develop faster than those that fed only on algae (Heinen & Abdella, 2005). Fowler's toad tadpoles, on the other hand, are known to exhibit reduced survival to metamorphosis when in the presence of tadpoles of other species, including Gray Treefrogs (*Dryophytes chrysoscelis*) and Coastal Plain Toads (*Incilius nebulifer*) Parris & Cornelius, 2004; Vogel & Pechmann, 2010). Tadpoles of the closely related Woodhouse's Toad (*Anaxyrus woodhousii*) are known to take a longer time to reach metamorphosis and suffer lower survivorship when in the presence of larger tadpoles of the same species (Woodward, 1987). In addition, American toad tadpoles are known to have a higher survivorship, greater weight, and shorter time to metamorphosis when they are introduced to a mesocosm before other species (Alford & Wilbur, 1985), which may confer a fitness advantage over any co‐occurring Fowler's toad tadpoles.

It was expected that disadvantaged tadpole species, in this case the Fowler's toad, would have lower survivorship and a lower weight at metamorphosis (Bardsley & Beebee, 1998; Cabrera‐Guzmán et al., 2013; Griffiths, 1991). However, this is not what occurred, as the surviving Fowler's toadlets from mixed‐species mesocosms in 2018 were larger than their single‐species counterparts. It is possible that the reduced density of the surviving Fowler's toad tadpoles led to decreased intraspecific competition, resulting in their larger size. A similar result occurs when exposure to toxic Cane Toad (*Rhinella marina*) eggs decrease the survivorship, but increase the weight, of Ornate Burrowing Frog (*Platyplectrum ornatum*) metamorphs (Crossland et al., 2009). Fowler's toad tadpoles also had a lower weight at metamorphosis when raised with Gray Treefrog (*Dryophytes versicolor*) tadpoles than when raised alone (Parris & Cornelius, 2004). As well, Fowler's toad tadpoles grown at lower densities have been found to be larger as metamorphs, even when competitors are not present (Yagi & Green, 2016), indicating that intraspecific competition may be important in determining the growth of this species. Contrarily, when replicated in 2021, both Fowler's and American toadlets had a significantly lower weight at metamorphosis in mixed‐species mesocosms compared with single‐species mesocosms. As there was high survivorship in all mesocosms and thus no difference in tadpole density between single‐species and mixed‐species mesocosms, we can conclude that the lower weight of toadlets emerging from the mixed‐species mesocosms is likely due to intraspecific competition in 2021 and not diet or tadpole density.

Time to metamorphosis is also an indicator of fitness in toadlets, with toadlets emerging later being considered to have a lower fitness (Bardsley & Beebee, 1998; Griffiths, 1991). We noted that in single‐species mesocosms in 2021, toadlets emerging later in the season also tended to weigh less than their conspecifics who metamorphosed earlier from the same mesocosm. The mixed‐species mesocosms revealed a different trend, however. Not only did tadpoles metamorphose later in mixed‐species mesocosms, and only after most of the American toad tadpoles had completed metamorphosis, but they did not decrease in weight over time, a stark contrast to all other mesocosms (Figure 2). This could indicate that a competitive release occurred in 2021, allowing the Fowler's tadpoles to grow and reach metamorphosis only after the American toad tadpoles had left the mesocosms.

Notably, one higher fitness metric in toadlets may not compensate for another, lower one. Despite higher survivorship of Fowler's toad tadpoles in 2021 than 2018 when in mixed‐species mesocosms, their lower weight is still likely to be a detriment to their fitness, potentially leading to lower juvenile survival, lower reproductive success, and later age at reproduction (Semlitsch et al., 1988; Smith, 1987; Woodward, 1983). While some studies have found that newly metamorphed froglets can compensate for their small size with increased terrestrial growth (Bouchard et al., 2016), studies on Fowler's toads indicate that this species cannot (John‐Alder & Morin, 1990; Yagi & Green, 2018), and thus this disadvantage as toadlets is likely to carry‐over into adulthood.

The differences in species interactions between these 2 years have several possible causes. Phenological shifts in breeding time may impact competition outcomes, as tadpole developmental stage has been found to impact competition (Banks & Beebee, 1987) and the timing of tadpole hatching (Alford & Wilbur, 1985; Lawler & Morin, 1993; Rudolf, 2018). Inter‐annual variation also has the potential to alter pond ecosystems and species interactions. Year effects such as precipitation can alter the hydroperiod of ponds, shifting the interactions of species and of ecosystems (Reinhardt et al., 2015). Yearly changes in nutrient concentration or algal biomass in ponds could also result in different outcomes of competition and tadpole performance (Connelly et al., 2008).

Stable ecosystems are often the exception, and not the rule (Gómez‐Rodríguez et al., 2010; Ricklefs & Schluter, 1993), and thus species interactions can change with shifts in their ecosystem. In the small pond ecosystems that Fowler's and American toad tadpoles inhabit, zooplankton and algal communities fluctuate in abundance and community structure within and between years, either with obvious climatic and environmental changes or without them (Dakos et al., 2009; Florencio, Fernández‐Zamudio, et al., 2020). Tadpoles interact with zooplankton and algal communities, and changes in these communities or abundance would alter food availability to the tadpoles (Connelly et al., 2008; Hamilton et al., 2012; Winder & Schindler, 2004; Wood & Richardson, 2010), as well as the structure of their environment. Shifts in resources such as food sources can in turn alter the outcome of competition (Kupferberg, 1997; Rudolf & McCrory, 2018). With such changes in the surrounding aquatic communities and resources in lower guilds, it is logical that species interactions within the same guilds would also change, even within our semi‐controlled conditions.

This study is an example of a shift in species interactions based on yearly conditions. While very few field studies are replicated, a striking 76% of those that are show significant inter‐annual variation (Vaughn & Young, 2010). This inter‐annual variation should not be ignored, as stochasticity in pond environments can lead to higher biodiversity across the landscape (Chase, 2010) and alternate stable states (Chase, 2003, 2010). Inter‐annual variation can also result in changes in community assembly, leading to shifts in community structure and function (MacDougall et al., 2008; Manning & Baer, 2018; Sarremejane et al., 2018; Werner et al., 2020). While we are beginning to see the importance of yearly conditions in studies, especially in plant communities, studies considering year effects on animals, particularly vertebrates, is lacking (Werner et al., 2020).

We ran our study twice, in semi‐controlled conditions, and were still able to see drastically different results of species interactions. The effects of inter‐annual variation are often treated as noise in more long‐term studies, and overlooked (Rudolf, 2019). We show that a closer look at inter‐annual variation is warranted when examining species interactions, as changes in yearly conditions, precipitation, surrounding aquatic communities, and phenology can all alter the outcome of competing species. Year effects are likely to become even more pronounced in coming years, as climate change results in more extreme weather events, accelerating the need to consider and examine inter‐annual variation (Reinhardt et al., 2015; Rudolf, 2019; Stewart et al., 2019). It is necessary to replicate competition studies across years in order to obtain an improved understanding of how species may behave, develop, and interact differently in response to climatic changes and shifting conditions. Climate and environmental shifts such as habitat loss and food source variation are likely to alter competition dynamics in coming years, and studying inter‐annual variation allows for a glimpse into resulting ecological impacts. This study system thus provides an excellent opportunity to observe the interactions between inter‐annual effects and species competition, a dynamic relationship that holds potential relevance to a broad set of ecological systems.

## AUTHOR CONTRIBUTIONS

**Jessica Ford:** Conceptualization (lead); data curation (lead); formal analysis (lead); methodology (lead); writing – original draft (lead); writing – review and editing (equal). **David M. Green:** Supervision (lead); writing – original draft (supporting); writing – review and editing (equal).

## FUNDING INFORMATION

Funding was provided by the following sources: Fonds de Recherche du Québec—Nature et Technologies, Ontario Ministry of Natural Resources and Forestry, Natural Sciences and Engineering Research Council of Canada.

## CONFLICT OF INTEREST STATEMENT

The authors have no conflicts of interest to declare.

### OPEN RESEARCH BADGES

This article has earned an Open Data badge for making publicly available the digitally‐shareable data necessary to reproduce the reported results. The data is available at 10.5061/dryad.b8gtht7h9.

## Data Availability

Data are publicly available on DRYAD (doi:10.5061/dryad.b8gtht7h9).
